# A New Suture Material

**Published:** 1897-04

**Authors:** 


### A NEW SUTURE MATERIAL.

Dr. L. Aringino has prepared a new suture and ligature from the parietal peritoneum of cattle or horses. This is dissected off, cut into strips of different widths, twisted into cords and dried for two or three days. These strands are freed from fat by soaking in ether, and are made sterile by soaking in a two per cent. solution of corrosive sublimate in alcohol. They are preserved in a weaker solution of the same character. He claims that these sutures can be easily prepared, are strong, are absorbed without difficulty, and are easily sterilized. They are certainly less liable to contain bacteria and micro-organisms than the submucous layer of the intestine, which is the common form of material used as "catgut." Arangino also sterilizes his material by placing it in oil of juniper for ten minutes, heating to boiling, and preserves it in absolute alcohol.—*Medical Record.*

### PNEUMONIA IN CHILDREN.

Dr. Griffith (*The Medical News*, December 5, 1896) speaks as follows of this condition: "Among the conditions which render percussion difficult and unsatisfactory in childhood is the peculiar resiliency of the chest at this age. Unless the blow be light and percussion carefully performed, it is very easy to overlook a limited area of dullness which could easily have been found. It is as much or more the sensation of resistance to the finger which we must seek, as it is any alteration of the sound. Too hard a blow makes the whole chest reverberate, and prevents the possibility of local differentiation. I find a series of rapid, light blows the best method as a rule. But in spite of the greatest care it sometimes happens that a child will pass through an attack of pneumonia without at any time revealing a dullness on percussion. Indeed, it often happens that the affected side is tympanitic, rather than dull."

